# Cremation of the Dead

**Published:** 1889-12

**Authors:** 


CREMATION OF THE DEAD.

Two articles upon this interesting subject from the pen of Dr. Wm. B. Clarke, of Indianapolis, appear in the *Indianapolis Sentinel* of September 22d and October 6th. They are excellent and exhaustive articles and should be read by all who desire to know all that is said in favor of this great reform in the disposal of the dead.

